# Pharmacological and Therapeutic Applications of Esculetin

**DOI:** 10.3390/ijms232012643

**Published:** 2022-10-20

**Authors:** Sourbh Suren Garg, Jeena Gupta, Debasis Sahu, Chuan-Ju Liu

**Affiliations:** 1 Department of Biochemistry, School of Bioengineering and Biosciences, Lovely Professional University, Phagwara 144001, Punjab, India; 2 Department of Orthopedic Surgery, New York University Grossman School of Medicine, New York, NY 10010, USA

**Keywords:** esculetin, cancer, oxidative stress, inflammation, arthritis, diabetes, fatty liver, pharmacokinetic, chromatography

## Abstract

Esculetin is a coumarin compound, which belongs to the class of benzopyrone enriched in various plants such as *Sonchus grandifolius*, *Aesculus turbinata*, etc. Free radicals lead to the development of oxidative stress causing inflammation, arthritis, cancer, diabetes, fatty liver disease, etc. These further reduce the efficacy of anticancer drugs, activate inflammatory signaling pathways, degrade joints and cartilage, and disrupt the glycemic index and normal function of liver enzymes. For instance, the current treatment modalities used in arthritis such as non-steroidal anti-inflammatory drugs, disease-modifying anti-rheumatoid drugs, and lipoxygenase inhibitors present limited efficacy and adverse effects. Thus, there is a constant need to find newer and safer alternatives. Esculetin has an immense antioxidative potential thereby alleviating arthritis, diabetes, malignancies, and hepatic disorders. Structurally, esculetin contains two hydroxyl groups, which enhance its ability to function as an antioxidant by inhibiting oxidative stress in pathological conditions. Leukotriene B4 synthesis, NF-κB and MPAK pathway activation, and inflammatory cytokine production are the main causes of bone and joint deterioration in arthritis, whereas esculetin treatment reverses these factors and relieves the disease condition. In contrast, lipid peroxidation caused by upregulation of TGF-β-mediated expression and dysfunction of antioxidant enzymes is inhibited by esculetin therapy, thus reducing liver fibrosis by acting on the PI3K/FoxO1 pathway. Therefore, targeting NF-κB, pro-inflammatory cytokines, TGF-β and oxidative stress may be a therapeutic strategy to alleviate arthritis and liver fibrosis.

## 1. Introduction

**Figure 1:**
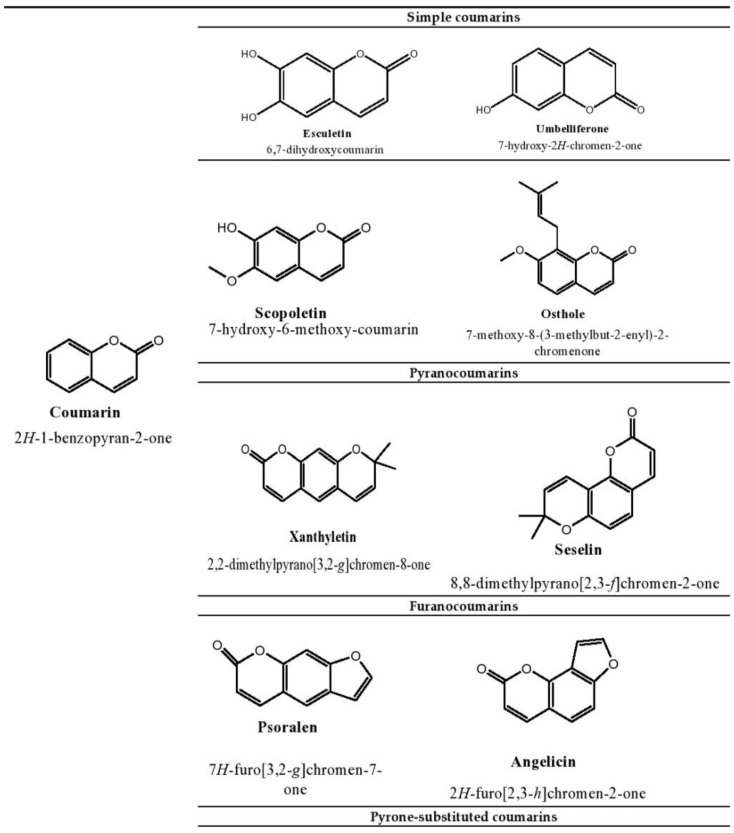
Structure of coumarin compounds.

The plant kingdom is the richest source of naturally occurring phenolic compounds, as there are about 8000 phenolic compounds in its distribution [1]. The presence of these phenolic compounds in nature has been extensively used by researchers to tackle disease stratification. Coumarins are one of the main bioactive phenolic or heterocyclic compounds with their large distribution in nature. These compounds belong to the benzopyrone class and are found in various plants, notably in the seeds of the tonka bean and *Dipteryx odorota*. These phenolic compounds are classified into four types, namely, simple coumarins, furanocoumarins, pyranocoumarins, and pyrone-substituted coumarin [2]. Umbelliferone, esculetin, scopoletin, and osthole are some examples of simple coumarin compounds. Coumarin derivatives that are hydroxylated and alkoxylated are classified as simple coumarins. The origin of furanocoumarins involves the association of the five-membered heterocyclic furan ring with coumarins, while their analogs are called pyranocoumarins, which are six-membered heterocyclic non-aromatic compounds containing five carbon atoms. Psoralen, angelicin, and xanthyletin are some examples of furanocoumarins, while seselin is a member of pyranocoumarins. Warfarin is a potent anti-coagulant derivative of coumarin and is classified in pyrone-substituted coumarins [2] (Figure 1).

Several reports have highlighted that some anticancer drugs used in the treatment of tumorigenesis not only kill malignant cells but also affect normal cells by producing reactive oxygen species that subsequently damage deoxyribonucleic acid (DNA), proteins, and lipids [3,4]. For example, doxorubicin is a potent anti-cancer drug that causes apoptosis in malignant cells by accumulating hydroxyl radicals, resulting in the development of oxidative stress in the body [5]. 2-Methoxyestradiol is another drug that exhibits anti-cancer properties by activating the c-Jun-N-terminal kinase (JNK) signaling pathway-induced apoptosis [6,7]. In addition, this drug has been found to induce hydrogen peroxide (H_2_O_2_) in mitochondria [8]. An in vitro study by Heo et al. reported that resveratrol induces cell-cycle arrest in melanoma cells (A375SM cells) by activating the reactive oxygen species (ROS)-mediated P53–P38 and ER stress pathway [9]. These studies have shown that drugs used in cancer therapy can act as a source of oxidative stress in the body.

Inflammation recruits the immune cells by liberating the chemokines and cytokines toward the site of oxidative stress [10]. Increased production of ROS by polymorphonuclear neutrophils at the site of inflammation leads to tissue injury and endothelial dysfunction [11]. Likewise, an increase in ROS and reactive nitrogen species increased the level of isoprostanes and prostaglandins (PGE), which in turn caused rheumatoid arthritis (RA) [12]. Published reports claim that patients with asthmatic symptoms have higher levels of ROS [13,14]. This demonstrated that the generation of free radicals can lead to degenerative diseases such as asthma.

The etiology of arthritis Is not completely understood [15]; however, it is implied that activation of nuclear factor-kappa B (NF-κB) and mitogen-activated protein kinase (MAPK) signaling pathways are major contributors to this disease. Phosphorylated IkB can cause NF-κB translocation to the nucleus in response to lipopolysaccharides (LPS) stimulation, where it binds to target genes and produces inflammatory cytokines [16,17]. Similarly, the release of pro-inflammatory cytokines such as tumor necrosis factor-α (TNF-α), interleukin (IL)-1, and matrix metalloproteinase (MMP) enzymes such as MMP-2, -3, and -9 cause cartilage and joint deterioration in arthritis [18,19]. Conversely, oxidative stress is thought to be a primary contributor to the persistent inflammation prevalent in arthritis cartilage. In osteoarthritis (OA), the level of inflammatory mediators such as IL-1β, TNF-α and IL-6 are increased, which directs the induction of reactive oxygen species and causes matrix breakdown and joint deformation. Since ROS and inflammation are interconnected, they become excellent therapeutic targets [20].

Besides cancer and inflammation, diabetic patients are found to have a low level of antioxidant enzymes that trigger a cascade of hyperglycemia-induced oxidative stress [21,22]. Diabetic nephropathy is one of the serious complications associated with diabetic individuals. Together, oxidative stress and inflammation alter lipid and protein functions and induce glycoxidation in diabetes [23,24], suggesting the role of ROS in diabetes and its associated complications.

Fat accumulation has been reported in individuals with non-alcoholic fatty liver conditions, and those with obesity, high blood sugar, and cholesterol are at great risk of developing such conditions [25,26,27,28,29,30,31,32]. In the liver, ROS such as superoxide anion radicals and H_2_O_2_ are present which promotes the pathogenesis of oxidative stress [33]. A high level of ROS leads to changes in the structure of proteins, lipids, and DNA, which consequently accumulates the ruptured macromolecules and causes liver injury such as fatty liver [34]. Conversely, high levels of by-products of lipid peroxidation (hydroxyoctadecadienoic acid and hydroxyeicosatetraenoic acid) are elevated in the liver, with an increase in triglyceride, suggesting the role of free radicals and high-fat diet (HFD) in the development of fatty liver by inducing the lipid peroxidation [35].

Carbon tetrachloride is a common agent for lipid peroxidation, which can cause liver damage [36]. Cytochrome P450 2E1 is an enzyme that enables the conversion of carbon tetrachloride to trichloromethyl radicals to initiate lipid peroxidation in the body and signals the lipids to produce oxidation products that ultimately hinder the functioning of the liver by developing a condition called liver injury [37].

These facts showed that apoptosis, imbalance of glycemic index, hepatic failure, and the generation of free radicals are highly correlated with each other. It is therefore imperative to interrupt the link between cancer, inflammatory disorders, arthritis, diabetes, and hepatic diseases from oxidative stress to prevent the stratification of diseases.

Esculetin (6,7-dihydroxychromen-2-one) is a coumarin derivative that structurally contains the two hydroxyl groups at the 6th and 7th carbon atoms. The presence of more hydroxyl groups in esculetin helps scientists to replace hydroxyl groups with any group to prepare a new derivative against disease outbreaks. The bioactivities and therapeutic applications of coumarin compounds and their derivatives depend on their structural arrangement. Free radicals act as a potent source for the pathogenesis of many diseases, whereas the presence of hydroxyl groups on esculetin makes this compound more efficient to act as an antioxidant by inhibiting the oxidative stress in disease conditions [38,39,40]. It has been found that the attachment of the hydroxyl group to phenolic compounds can effectively connect with free radicals [41]. These hydroxyl groups in phenolic compounds can also exhibit chelation with transient metals such as copper and iron [42]. Esculetin exhibits dual modulation of apoptosis, as well as anti-diabetic and anti-inflammatory action that may be partly attributed to its antioxidant characteristic.

This coumarin compound is also known to have hepatoprotective properties, which can be attributed to its antioxidant properties. A study by Tien et al. showed that esculetin treatment at 100 and 500 mg/kg can scavenge free radicals generated during lipid peroxidation by improving the levels of antioxidant enzymes such as catalase (CAT) and superoxide dismutase (SOD). Similarly, the activity of liver enzymes such as alanine transaminase (ALT) and aspartate transaminase (AST) was also decreased upon esculetin treatment. Esculetin therapy with the same dose restricted cytochrome c release from mitochondria by increasing the levels of anti-apoptotic proteins such as B-cell lymphoma-2 (Bcl-2) and decreasing the levels of pro-apoptotic proteins such as truncated Bid (t-Bid) and Bcl-2. Therefore, this study provides a link that free radical scavenging is an important preventive approach to protect against liver disorders [43]. In another study, Lee et al. found that esculetin reduced oxidative stress in rat liver lesion models, which was evident in reduced necrosis, leukocyte infiltration, and edema of liver cells [44].

Furthermore, esculetin is the only coumarin derivative to demonstrate the vast array of biological activities, such as the inhibition of free radical generation, inflammatory markers, influencing of glycemic index, and abnormal functioning of genes in cancer and hepatic failures. These characteristics of esculetin make it unique compared to other coumarin derivatives.

## 2. Therapeutic Applications of Esculetin

Oxidative stress plays an important role in the pathogenesis of chronic ailments such as cancer, diabetes, liver disease, and inflammatory disorders. Esculetin exhibits numerous therapeutic applications such as free radical scavenging, suppression of dysregulated transcription factors in cancer, inhibition of inflammatory pathways involved in arthritis, management of glycemic index, fatty liver disease, etc.

### 2.1. The Role of Esculetin in Cancer Treatment

Cancer is a lethal condition that arises as a result of abnormal cell proliferation in the body and leads to organ failure and death. Many developments have been made either to control or cure this lethal disease. Coumarin compounds have always been considered the first choice for researchers because of their excellent biological activity and low toxicity [45]. A study by Arora et al. showed that esculetin at 100 µM arrests the growth of cancer cells in the G1 phase of the cell cycle. It impedes the binding interaction between nuclear factor erythroid 2-related factor 2(Nrf2) and Klech-like-ECH-Associated Protein-1 (KEAP-1) by activating the antioxidant response element (ARE) pathway and attenuating the NF-κB activity leading to apoptosis in the human pancreatic cancer cell (PANC-1) [46]. Esculetin at 20 µM inhibits leukemia cell proliferation and induces autophagy through the formation of autophagic vesicles. In addition, it downregulates the expression of cyclin D1, D3, DK4, and DK2, causing a cell-cycle arrest at the G0/G1 phase. This coumarin compound was shown to block the phosphorylation of MAPK and extracellular signal-regulated kinase (ERK), thereby inhibiting the activation of Raf/MAPK/ERK signaling [47], and was also reported to downregulate the JNK and ERK pathways in leukemia U937 cells at 30 µg/mL, suggesting its potential in inhibiting the tumorigenesis [48].

Benzo[a]pyrene is known to play an important role in lung cancer [49], and 50 mg/kg esculetin treatment showed an arrest in the cancerous cell proliferation by downregulating Bcl-2 and NF-κB, causing apoptosis [50]. Esculetin at 55 µg/mL concentration not only inhibits pancreatic and lung cancer but also prevents colon cancer by activating MAPK signaling pathways, caspase-3 and 9, leading to apoptosis. It was also found to release cytochrome c into the cytosol by increasing the depolarization of the mitochondrial membrane and increasing the B-cell lymphoma-2-associated x protein expressions [51]. In the oral squamous cancer cell, 20 µg/mL of esculetin has been shown to downregulate the expression of specificity protein 1 (Sp1), p27, cyclin D1, Mcl-1, and survivin, thus inducing apoptosis [52]. In larynx cancer, 2 and 10 µM esculetin treatment was found to inhibit the Janus kinase–signal transducer and activator of transcription (JAK/STAT) pathway by suppressing the phosphorylation of STAT3 and subsequent translocation into the nucleus. Furthermore, esculetin causes cell-cycle arrest at the G1/S phase and thus supports apoptosis [53].

**Figure 2:**
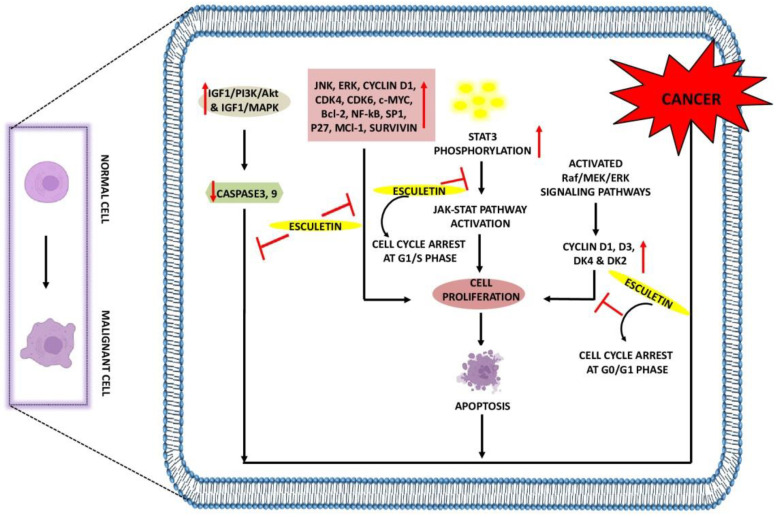
Anti-cancer effects of esculetin: Esculetin downregulates the IGF1/PI3K/AKT, IGF1/MAPK, JNK, ERK, NF-κB, P27, Survivin, and Bcl-2. Esculetin also inhibits cell proliferation by inhibiting the STAT3 phosphorylation and cyclin D1, D3, DK4, and DK2, leading to apoptosis and suppression of cancer. ↑: Overexpression; ↓: low expression; ⊣: inhibition.

In hepatocellular carcinoma, esculetin at 2.24 µM promotes apoptosis by arresting the cells at the S-phase of the cell cycle. In addition, esculetin was found to elevate the mechanism of caspase-3 and 9 and reduced the mitochondrial membrane potential. Esculetin significantly increased the Bax expression, thereby reducing the Bcl-2 expression, thus exhibiting its anti-cancerous potential [54]. Overactivation of insulin-like growth factor-1/phosphoinositide-3-kinase/Protein kinase B (IGF1/PI3K/Akt) and IGF1/MAPK signaling pathways contributed to the development of tumors; however, esculetin at 850 µM was found to diminish the mitochondrial membrane potential while simultaneously activating the mitochondrial apoptotic pathway in the MGC-803 gastric cancer cell line. It increased the cytochrome c release from mitochondria, Bax/Bcl-2 index, and activated the activity of caspase-3 and 9 by suppressing the IGF-1/PI3K/Akt pathway [55]. Esculetin at 200 µg/mL was reported to prevent the proliferation, migration, and invasion of renal carcinoma cells by cell-cycle arrest at G0/G1 and G2/M phase, downregulating the expression of Cyclin D1, CDK4, CDK6, and cellular myelocytomatosis (c-Myc) resulting in apoptosis. Levels of E-cadherin were increased, whereas the expressions of N-Cadherin and vimentin were downregulated with esculetin treatment [56] (Table 1, Figure 2).

### 2.2. The Role of Esculetin in Oxidative Stress Treatment

Free radicals are the single unpaired electron species that exist independently in the body and are capable of causing oxidative damage to DNA, proteins, and carbohydrates. Therefore, antioxidants act as an eminent tool against oxidative damage. Many coumarin compounds, including esculetin, have been evaluated using in vitro and in vivo models of oxidative stress. H_2_O_2_ is known to play a crucial role in generating oxidative stress. Treatment of 5 µM esculetin has been reported to increase the phosphorylation ofNrf2 and the expression of NAD(P)H:quinone oxidoreductase1(NQO1) in C2C12 myoblasts cells. In addition, esculetin at the same dose was also reported to activate the ERK signaling pathway and exhibit protective effects against H_2_O_2_-induced free oxidative stress [57]. Likewise, Kim and colleagues showed that Chinese hamster lung fibroblast cells (V79-4 cells) treated with H_2_O_2_ showed an increase in lipid peroxidation free radical generation, leading to DNA damage, whereas the free radical scavenging and intracellular ROS scavenging activity of esculetin were found to be 77% and 75% at 10 µg/mL [58]. Increased serum levels of alkaline phosphatase (ALP), AST, and ALT in carbon tetrachloride-induced injury in rat liver were reduced with 35 mg/kg esculetin dosage, which was mainly due to its lipid peroxidation and free radical inhibition activity [59].

**Figure 3:**
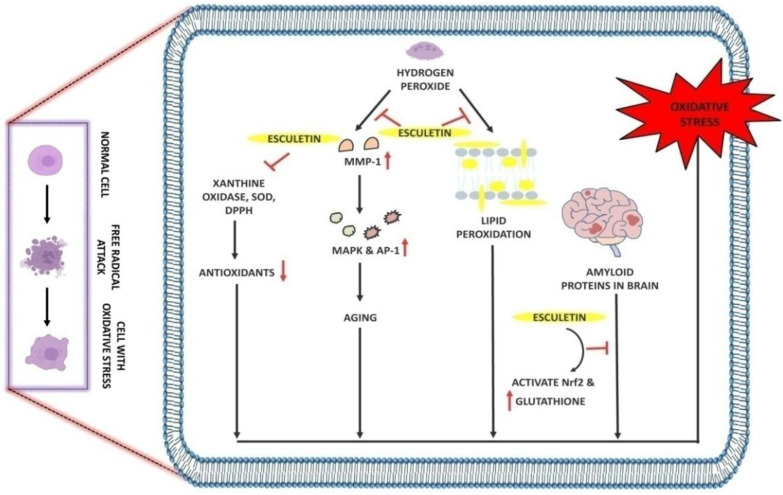
Antioxidant effect of esculetin: Esculetin blocks the activity of H_2_O_2_ which further inhibits the MMP-1 and lipid peroxidation in cell membranes. Esculetin prevents the brain from amyloid-induced oxidative stress via activating the Nrf2 and increasing the levels of glutathione, thus inhibiting oxidative stress. ↑: Overexpression; ↓: low expression; ⊣: inhibition.

In Alzheimer’s disease, 20 µM esculetin activates the Nrf2 and increases the glutathione levels in SH-SY5Y cells. In addition, esculetin increases the phosphorylation of ERK and Akt and thus protects cells from oxidative stress-induced damage by amyloid proteins [60]. In human dermal fibroblast cells, esculetin at 0.6 and 2.1 µg/mL concentrations effectively scavenges the superoxide and DPPH radicals and inhibits MMP-1 [61]. It has been found that H_2_O_2_ upregulates the expression of MMP-1, thus promoting skin aging and oxidative stress by activating the MAPK and AP1 signaling pathways. The treatment of 5 µg/mL esculetin to H_2_O_2_-induced oxidative stress in HaCaT cells results in the inhibition of MMP-1, phospho-MEK1, phospho-ERK1/2, phospho-SEK1, and phospho-JNK1/2 along with the intracellular Ca^2+^ levels [62]. In addition, esculetin at 50 µL was reported to protect human fibroblast from oxidative stress-induced DNA damage induced by linoleic acid hydroxide and iron (III) ion [63] (Table 2, Figure 3).

### 2.3. The Role of Esculetin in Inflammation Treatment

Inflammation is defined as the defense mechanism that occurs in response to infection or injury and helps the body to maintain homeostasis. During infection or injury, the damaged cell triggers the release of various physiological messengers including histamine, PGE, nitric oxide (NO), and leukotrienes, which promote the cascade of inflammation [64], resulting in the development of debilitating diseases such as RA and OA [65]. Much research has been conducted to reveal the molecular mechanism of esculetin as a potent inhibitor of inflammation.

The transcription factor NF-κB is involved in the pathogenesis through the regulation of inflammatory genes [66]. Briefly, the cytosol is a primary site of occurrence of NF-κB and under inflammatory stimuli; IκB is phosphorylated as well as degraded by the 26S proteasome resulting in the release of NF-κB. Consequently, the released NF-κB translocates to the nucleus, resulting in its binding to the promoter region and upregulation of inflammatory genes. A study by Yao-Jun et al. reported that esculetin at 20, 40 and 60 mg/kg downregulates the expressions of inflammatory cytokines and chemokines such as TNF-α, IL-1β, IL-6, CCL2, and inducible nitric oxide synthetase (iNOS). They also highlighted that the same dose of esculetin inhibits the NF-κB, STAT1, STAT3 and p65 expressions in LPS-induced macrophages and septic mice. In addition, the translocation of p65 from the cytoplasm to the nucleus was also inhibited upon esculetin treatment in macrophages. The phosphorylation of IKKα/β, IKBα, ERK1/2, JNK, and JMJD3 was also observed to downregulate in esculetin-treated LPS-induced macrophage cells [67]. Esculetin with a 12 µg/mL dose potentially inhibits the TNF-α, IL-1β, ROS, and LPS-mediated nuclear translocation of NF-κB p65 by suppressing the IKβ-degradation in RAW 264.7 cells [68]. A study by Jayakumar et al. found that esculetin reverse LTA-induced IkB degradation and NF-κB p65 phosphorylation. By increasing Nrf2 and scavenging DPPH radicals in RAW 264.7 cells at a concentration of 20 µM, esculetin inhibited the translocation of NF-κB p65 to the nucleus [69].

NO is one of the key mediators of inflammation [70]. Nitric oxide synthase (NOS) is an enzyme that exists in three different forms: neuronal NOS (nNOS), endothelial NOS (eNOS), and iNOS. This class of enzyme is responsible for the conversion of L-arginine into NO [71]. But when there is an imbalance in this reaction, NO levels rise, resulting in exacerbated inflammation. A study by Zhu et al. reported that 20 and 40 mg/kg doses of esculetin result in the inhibition of TNF-α, IL-1β, and IL-6 expressions in serum and hippocampus. Further, the downregulation of cyclooxygenase (COX)-2 and iNOS was also observed when treating the mice with esculetin. In addition, this coumarin compound with the same dose was also found to inhibit the LPS-induced pIKK-α, pIKK-β, p-IKB-α, and p-NF-κB65 activation. Esculetin also results in the activation of brain-derived neurotrophic factor/tropomyosin receptor kinase B (BDNF/TrKB) signaling pathway, by which the levels of p-TrKB protein were upregulated in serum and hippocampus, and thus, esculetin exhibits the neuroprotective activity [72]. Similarly, the treatment of esculetin at 80 and 120 µM to RAW 264.7 cells and BALB/c mice results in an increase in endocytic activity and augmented NO and iNOS levels in LPS-treated macrophages [73]. At 100 mg/kg and 200 mg/kg, esculetin inhibits the inflammatory cascade by attenuating the NO and PGE2 levels in synovial fluid and MMP-1 expression in the cartilage of osteoarthritic rabbits [74].

In the adipose tissues of obese mice, macrophages are known to release potent inflammatory agents, especially NO, PGE, and TNF-α causing systemic inflammation. Administration of a 100 µM dose of esculetin upregulates the level of heme oxygenase 1 in cocultured macrophages and adipocytes by restricting the liberation of TNF-α, NO and MCP-1. Moreover, the inhibition in peroxisome proliferator-activated receptor (PPAR)-ϒ and CCAAT/enhancer binding protein α was also reported to inhibit with esculetin treatment, suggesting its protective role against the obesity-induced inflammation [75]. Proinflammatory cytokines such as TNF-α, IL-1β, IL-2, interferon (IFN)-γ, IL-8, and IL-6 are produced during macrophage activation and leads to the inflammatory diseases [76]. Both doses of esculetin (100 µM and 5 mg/kg) were found to inhibit the production of TNF-α, IL-1β, IL-2, INF-ϒ in rats and RAW 264.7 cells. Esculetin was also reported to prevent the generation of ROS and reduced glutathione (GSH) depletion. Similarly, the activities of myeloperoxidase (MPO) and ALP were inhibited with esculetin treatment [77].

A study by Singh et al. highlighted the protective effects of esculetin in reserpine-induced fibromyalgia in female Swiss albino mice. They found that esculetin at 10 mg/kg effectively increases the GSH and brain serotonin (5-HT). In addition, the levels of TNF-α, IL-1β, and thiobarbituric acid reactive substances (TBARS) were found to decrease in the brain with esculetin treatment [78]. On the contrary, the inhibition of the NF-κB signaling pathway by esculetin (10, 20 and 40 µmol/L) prevents the release of histamine-induced IL-6, IL-8, and MUC5AC in human nasal epithelial cells. Furthermore, the suppression in histamine-induced p-p65 expression and p-IKBα was also found to decrease with esculetin treatment [79]. Esculetin isolated from *Fraxinus rhynchophylla* was found to attenuate the expressions of TNF-α/IFN-ϒ-induced phosphorylation of STAT1 and NF-κB(p65) translocation by degrading the IKBα at 2, 10, 50 mg/kg and 10 µM doses. Treatment with esculetin was also reported to decrease the expressions of IL-4, IL-13, IL-31, and IL-17A. Similarly, the thickness of the epidermal and dermal ear, infiltration of eosinophil, and accumulation of mast cells were found to be reduced with esculetin treatment in 2-4-dinitrochlorobenzene/dermatophagoidesfarinae extract (DNCB/DFE)-induced acute skin inflammation model [80].

Esculetin at 5 µM concentration effectively reduces the LPS-induced phosphorylation of ERK1/2 and NF-κB expressions and protects the cells from apoptosis and necrosis as well. Moreover, reduction in levels of LPS-induced TRAIL, TNFR, IL-1β, IL-6, IL-12, vascular epidermal growth factor (VEGF), Manganese superoxide dismutase (MnSOD), and glutathione peroxidase (GPx) was observed with esculetin treatment in LPS-induced inflammation in human retinal pigment epithelial cells (ARPE-19 cells) [81]. Both doses of esculetin (20 and 40 mg/kg, 0.1, 1, and 10 µM) were reported to inhibit the MPO activity, neutrophil infiltration, NF-κB pathway, RhoA/Rho kinase pathway, followed by the downregulation of TNF-α, IL-1β, and IL-6 in A549 cells and BALB/c mice [82]. The TNBS-induced colitis in HCT116 cells and Sprague Dawley colitic rats was found to ameliorate with esculetin treatment at 100 and 200 µM doses. It inhibits MPO, COX-2, and iNOS expression and activates the HIF-1 in HCT116 cells, which results in the activation of hypoxia-inducible factor (HIF)-1α and VEGF. Furthermore, esculetin was also reported to prevent the activation of HIF-prolyl hydroxylase-2enzyme and thus exhibits anti-colitic effects [83].

**Figure 4:**
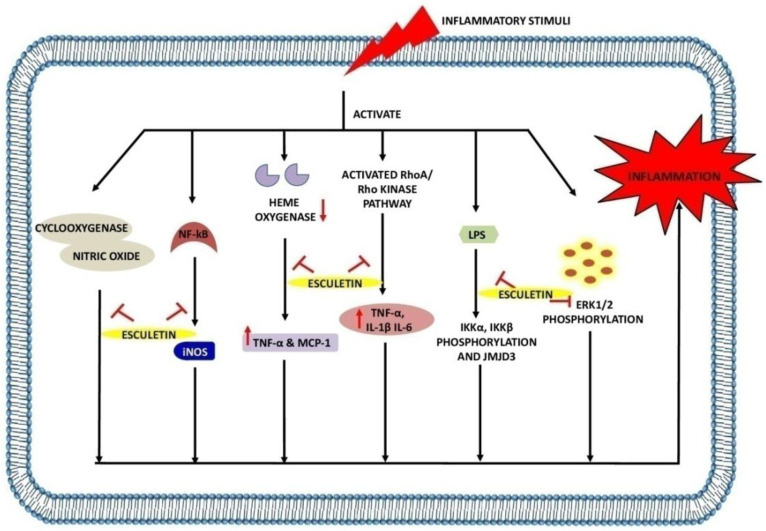
Anti-inflammatory effects of esculetin: Esculetin inhibits the action of cyclooxygenase, NF-κB, NO, LPS, heme oxygenase, RhoA/Rho kinase pathway, and ERK1/2 phosphorylation, inhibiting the inflammatory reactions. ↑: Overexpression; ↓: low expression; ⊣: inhibition.

A study by Chen et al. highlighted the anti-psoriatic effects of esculetin in imiquimod-induced psoriasis in BALB/c mice. They showed that esculetin at 50 and 100 mg/kg doses significantly ameliorate the skin lesions by decreasing the expressions of TNF-α, IL-6, IL-22, IL-23, IL-17A, and IFN-γ cytokines. Moreover, repression of phospho-IKKα and phospho-p65 was also observed with the inhibition of the NF-κB signaling pathway. The reduction in effector CD8+ T cells and upregulation in CD4+ FOXp3+ Treg frequency in spleen and lymph nodes results in amelioration of skin disease with esculetin therapy. This coumarin compound decreased the Ki67 and K10 mRNA expressions and the CD3+ and CD8+ T-cell infiltration in psoriatic mice [84] (Table 3, Figure 4).

### 2.4. The Role of Esculetin in Arthritis Treatment

Arthritis is characterized by acute or chronic inflammation of the joints accompanied by severe pain and structural damage. The two most frequent types of arthritis that afflict the global population are OA and RA [85]. OA is a degenerative disease characterized by progressive cartilage loss and bone deterioration [86], whereas RA is a systemic, persistent inflammatory condition driven by an autoimmune response to stimuli in the environment mainly affecting the synovial joints [87]. Rapid loss of articular cartilage, degradation of collagen and proteoglycans, upregulation of matrix metalloproteinases—leukotrienes (particularly leukotriene B4)—and thickening of the subchondral plate are the main contributors to the OA and RA [88,89,90,91,92,93,94]. A study by Yamada et al. reported that MMP-1, MMP-3, matrix degradation, and IL-1α-induced release of proteoglycans in cartilages of osteoarthritic rats was inhibited with esculetin treatment (10–100 µM) [95]. Similarly, esculetin at 100 µM significantly inhibits proteoglycan depletion, pro-MMP1, pro-MMP3, and MMP production in rabbit chondrocytes [96]. Esculetin at 10 mg/kg dose effectively reduces the level of leukotriene B4 in the plasma of rats with adjuvant-induced arthritis (AIA) [97].

**Figure 5:**
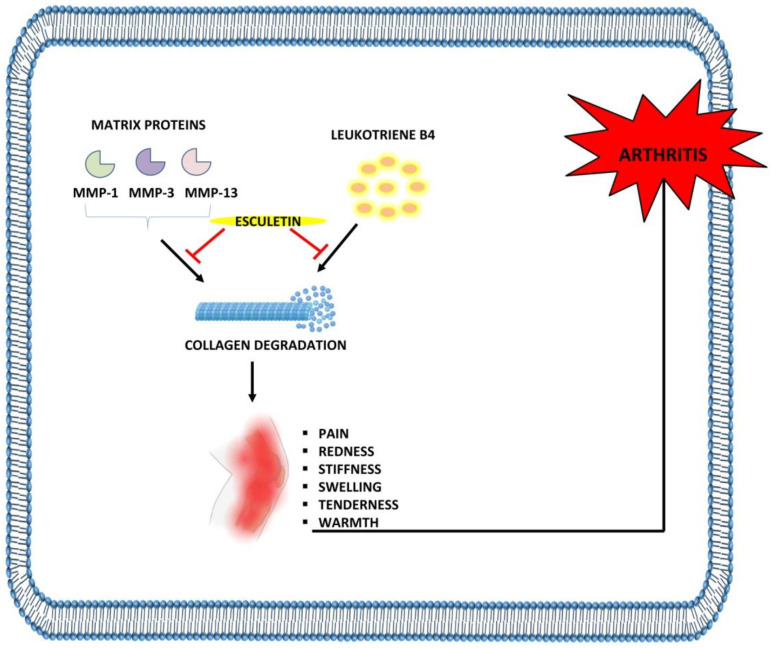
Anti-arthritic effects of esculetin: Esculetin inhibits the degradation of cartilage through the suppression of MMP-1, MMP-3, MMP-13, and leukotriene B4, leading to the inhibition of arthritis. ⊣: inhibition.

An esculetin derivative, 4-Methylesculetin at 50 mg/kg was reported to improve inflammation by decreasing the expression of TNF-α, IL-1β, IL-6, COX-2, PGE2 which directly inhibits the swelling and cartilage destruction. Its administration also downregulates the expression of bone-degrading enzymes such as cathepsin D (Cat D), acid phosphatase (ACP), ALP, and tartrate-resistant acid phosphatase (TRAP). It also inhibits inflammation-induced oxidative stress by preventing the endogenous generation of ROS. The restoration in the levels of antioxidant enzymes such as SOD, glutathione-s-transferase (GST), CAT, and liver enzymes such as AST, ALP were also restored with 4-methylesculetin therapy. This compound was also found to attenuate the NF-κB and Akt signaling pathway in the AIA model and thereby exhibits anti-inflammatory potential [98]. Another study by Elliott et al. highlighted that esculetin (66 µM, 100 µM, and 50 µmol/L) significantly reduced the MMP-1, MMP-3, and MMP-13 expression induced by IL-1α with oncostatin M. In addition, administration of esculetin also results in the inhibition of proteoglycan and collagen resorption in T/C28a4 cells, suggesting the protective role of esculetin in RA and OA [99] (Table 4, Figure 5).

### 2.5. The Role of Esculetin in Diabetes Treatment and Its Associated Complication

Diabetes is a metabolic disorder in which changes in plasma levels of glucose occur as a result of defects in insulin secretion or insulin action [100]. In such conditions, the activity of several antioxidant enzymes is found to be downregulated, resulting in the development of diabetes-induced oxidative stress [45,101,102]. Many natural and synthetic compounds have been used to ameliorate oxidative stress, and esculetin is one of them.

Administration of esculetin at 40 mg/kg was reported to reduce blood glucose levels and increase plasma insulin levels in streptozotocin (STZ)-induced diabetes in male albino rats. Moreover, esculetin restores the level of antioxidant enzymes, particularly GST, SOD, CAT, GPx, TBARS, lipid hydroperoxides, conjugated dienes, vitamin c, tocopherol and GSH in liver and kidney tissues of diabetic rats [103]. As STZ and an HFD result in an increase in triglyceride and plasma glucose concentrations, esculetin at 50 and 100 mg/kg improves the insulin sensitivity and reduces systolic blood pressure under in vivo hyperinsulinemic conditions. In addition, the increase in angiotensin II type 1 and 2 receptor expressions were prevented by esculetin. Treatment of this compound was also found to attenuate the vascular hyper-responsiveness to angiotensin II and impair acetylcholine-mediated relaxation with the downregulation of transformin growth factor-β (TGF-β) and KEAP-1 expression [104].

Esculetin not only reduces blood glucose levels and diabetes-mediated oxidative stress but also lessens the associated complications such as diabetic nephropathy, suggesting the role of esculetin as an anti-diabetic compound. A study by Surse et al. highlighted that administration of esculetin (50 and 100 mg/kg) results in reducing the level of TBARS, blood glucose, blood urea nitrogen, and plasma creatinine with the increase in plasma albumin levels in STZ-induced type I diabetic nephropathy in Sprague Dawley rats. They also reported that esculetin therapy blocks the TGF-β1-mediated fibronectin expression by attenuating the downregulation of PPARϒ expressions in diabetic nephropathic rats. In addition, epigenetic studies of this study showed a decrease in Bmp6 and increase in Mmp13 expression, which concludes the potential of esculetin in ameliorating the diabetes and kidney disease associated with it [105].

**Figure 6:**
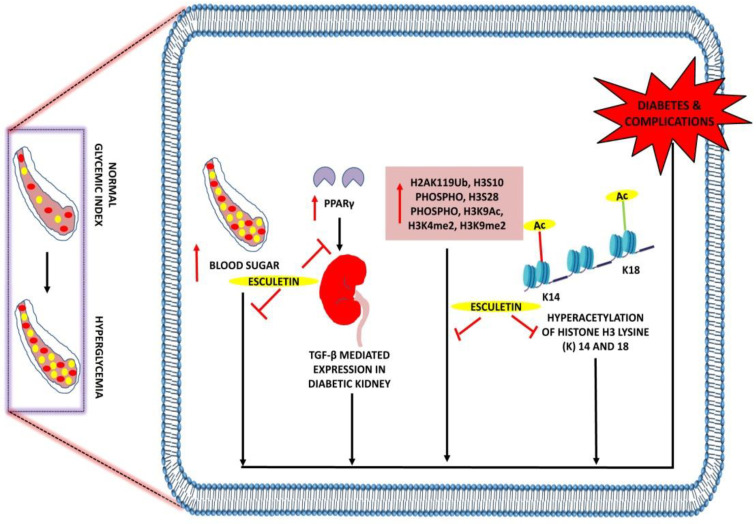
Anti-diabetic effects of esculetin: Esculetin downregulates the expression of PPARγ, H3S10phospho, H3S28phospho, H3K9Ac, H3K4me2, and H3K9me2, hyperacetylation of histone H3 lysine (K) 14 and 18 and attenuates diabetes and its associated complications. ↑: Overexpression; ⊣: inhibition. •: RBC; •: Sugar.

At the molecular level, various modulated epigenetic markers such as H3S10phospho, H3S28phospho, H3K9Ac, H3K4me2, and H3K9me2 are responsible for the pathogenesis of insulin resistance and type 2 diabetes. Treatment of esculetin at 50 and 100 mg/kg dose was found to decrease the plasma glucose, triacylglycerol, total cholesterol and systolic blood pressure levels in insulin resistance and type 2 diabetic rats. Furthermore, the expressions of angiotensin II type 1 and 2 receptor, Ki67, KEAP-1 were found to decrease with an increase in angiotensin-converting enzyme 2 (ACE2) expressions in insulin resistance and type 2 diabetic rats after receiving the esculetin therapy. This compound was also observed to prevent the cardiac fibrosis and cardiac hypertrophy in heart tissues of diabetic rats. The epigenetic results of this study showed that there was a decrease in the expressions of H2AK119Ub and H2BK120Ub when diabetic rats were treated with esculetin. This concludes that esculetin can potentially reversed these modified epigenetic markers and decrease the risk of type 2 diabetic cardiomyopathy [106]. Administration of esculetin at 50 and 100 mg/kg to diabetic nephropathic rats significantly improves the SOD1, GSH, angiotensin II receptor type I and ACE2 enzyme. In addition, the expressions of Mcp1, TGF-β and H2AK11Ub were found to downregulate in diabetic kidney, suggesting its potential in ameliorating the insulin sensitivity, hyperglycemia and renal dysfunction [107] (Table 5, Figure 6).

### 2.6. The Role of Esculetin in Hepatic Failure Treatment

Non-alcoholic fatty liver disease is becoming the primary cause of chronic liver disease due to the increasing evidence of obesity and type 2 diabetes mellitus. A study by Pandey et al. showed that both doses of esculetin (50 and 100 mg/kg) improve the phospho-Forkhead box protein O1 (FOXO1) expression by acting on the Akt/P13K/FOXO1 pathway, which further results in the amelioration of TGF-β-mediated hepatic fibrosis in HFD fed rats. In addition, the levels of AST, ALT and GSH were increased, whereas plasma triglycerides, cholesterol, and insulin were decreased in fatty rats. The same dose of esculetin was found to prevent the accumulation of extracellular matrix proteins [108].

**Figure 7:**
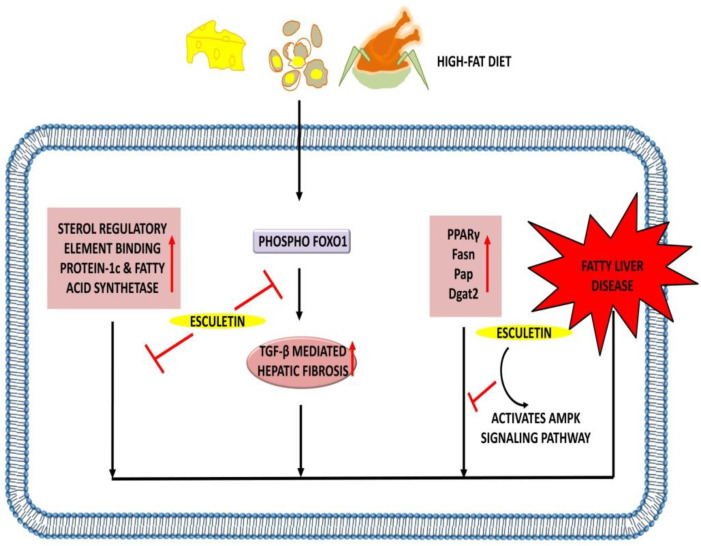
Hepatoprotective effects of esculetin: Esculetin downregulates the expression of SREBP1c, fatty acid synthetase, and TGF-β-mediated hepatic fibrosis. Esculetin also activates AMPK signaling pathway and inhibits the action of PPARγ, Fasn, Pap, and Dgat2, thus inhibiting the pathogenesis of the fatty liver disease. ↑: Overexpression; ⊣: inhibition.

The upregulation of PPARγ, Fasn, Pap, and Dgat2 is actively responsible for the development of non-alcoholic fatty liver disease in diabetes. Esculetin (0.01% *w*/*w*) exhibits hepatoprotective activity by downregulating the level of genes responsible for hepatic fatty acid and triglyceride synthesis [109]. Esculetin (25, 50 and 100 µM) dosages significantly decreased the expression of sterol regulatory element-binding protein-1c (SREBP1c) and fatty acid synthase through the activation of adenosine monophosphate-activated protein kinase (AMPK) signaling pathway. Furthermore, the phosphorylation of AMPKα (Thr 172) and ACC (Ser79) were increased in HepG2 cells upon esculetin therapy [110]. The results of all these studies prove the ability of esculetin in attenuating the development of non-alcoholic fatty liver disease (Table 6, Figure 7).

## 3. Synthesis of Esculetin

Esculetin is present naturally in the *Cortex Fraxini*; however, researchers have attempted to synthesize the esculetin in vitro. Many studies have been proposed to describe the chemical reactions resulting in the synthesis of esculetin and its related derivatives.

**Figure 8:**
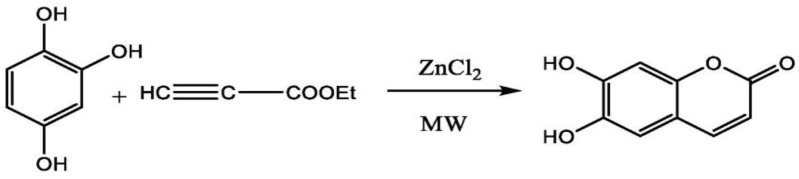
Synthesis of esculetin using microwave irradiation method.

A study by Yang et al. carried the synthesis of esculetin using microwave irradiation method. ZnCl2 is used as a catalyst to mediate the reaction. Briefly, they used the 1,2,4-benzentriol, ethyl propionate and ZnCl_2_ in the ratio of 1:1:3.5 g. They ran this reaction at a temperature of 105 °C for 10 min and adjusted the microwave power to 400 W. Under optimal conditions, these parameters gave a final yield of esculetin of around 87.4% [111] (Figure 8).

**Figure 9:**
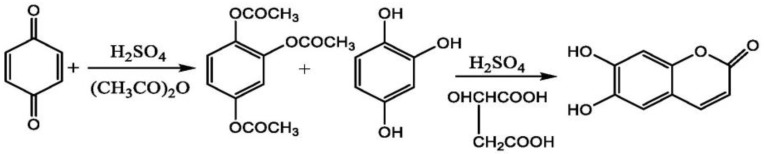
Synthesis of esculetin using p-benzoquinone, sulfuric acid and acetoacetate.

In another study, Zhang et al. performed the chemical synthesis of esculetin using raw materials. The reaction started with the addition of concentrated sulfuric acid and acetoacetate to p-Benzoquinone in the ratio of 0.15:3:1, at 45 °C. The solution was allowed to stir continuously for 3 h, which resulted in the formation of peracetylated 1,2,4-benzenetriol. The product formed was then catalyzed by concentrated sulfuric acid and malic acid with an equivalent amount of 1,2,4-benzenetriol, which resulted in the formation of esculetin as an end-product of this reaction. The final yield of esculetin obtained after this chemical reaction was 80% [112] (Figure 9).

**Figure 10:**
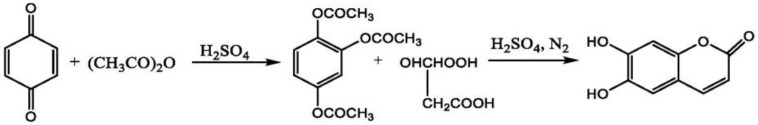
Synthesis of esculetin using p-benzoquinone, sulfuric acid and acetic anhydride.

A group of researchers showed the mechanism of esculetin synthesis and the final yield obtained after the reaction. The reaction started with the addition of sulfuric acid to p-Benzoquinone and acetic anhydride, which forms the 1,2,4-phloroglucinol triacetate. The product formed in the last reaction was treated with malic acid and concentrated sulfuric acid, which resulted in the formation of esculetin as an end-product of this reaction. The final yield of esculetin obtained after this chemical reaction was 80.3% [113] (Figure 10).

**Figure 11:**
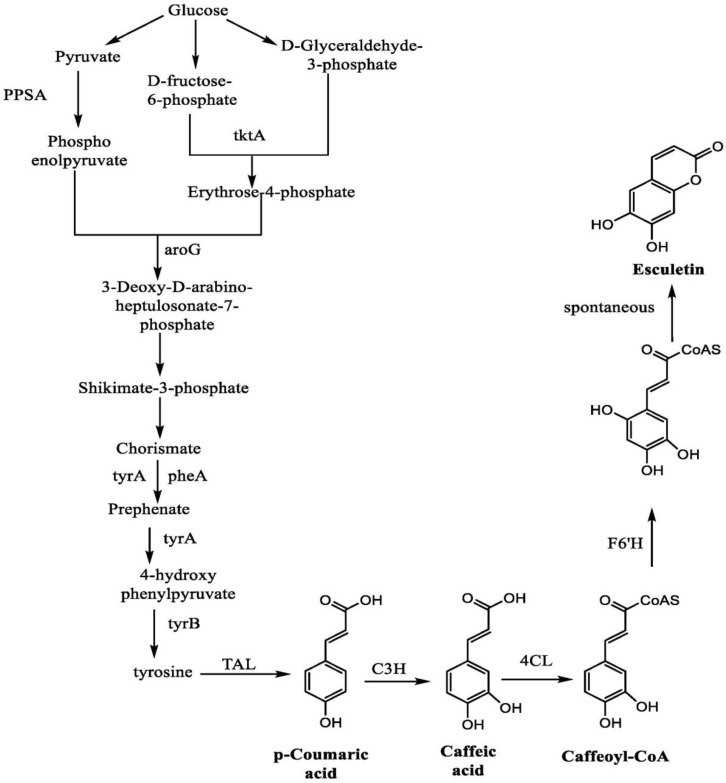
Synthesis of esculetin from glucose.

In a study, Yang et al. highlighted the synthesis of esculetin from glucose with the help of many intermediate steps. The reaction starts with the conversion of glucose into pyruvate, D-fructose-6-phosphate, and D-glyceraldehyde-3-phosphate. Pyruvate results in the formation of phosphoenol pyruvate with the help of an enzyme called phosphoenolpyruvate synthetase (PPSA). Similarly, D-fructose-6-phosphate and D-glyceraldehyde-3-phosphate form the erythrose-4-phosphate with the action of transketolase enzyme (tktA). Both of these products form 3-deoxy-D-arabino-heptulosonate-7-phosphate with the activity of deoxyphosphoheptonate aldolase (aroG) enzyme, which further leads to the formation of shikimate-3-phosphate and chorismate. The reaction of chorismate with prephenate dehydrogenase (tyrA) and prephenate dehydrate (pheA) forms the prehenate. In addition, this will lead to the formation of 4-hydroxy-phenylpyruvate, with an enzyme called prephenate dehydrogenase. Tyrosine is formed from the 4-hydroxy-phenylpyruvate with phenylalanine aminotransferase (tyrB) enzyme. The action of tyrosine amino lyase (TAL) enzyme on tyrosine catalyzes the formation of p-coumaric acid. An enzyme called coumarate-3-hydroxylase (C3H) mediates the conversion of p-coumaric acid into caffeic acid which further forms the caffeoyl-CoA with the help of 4-coumaroyl-CoA (4CL) enzyme. Caffeoyl-CoA with feruloyl-CoA6′-hydroxylase (F6′H) forms one intermediate product, which mediates the formation of esculetin as an end-product to this reaction [114] (Figure 11).

## 4. Detection, Pharmacokinetic and Metabolic Studies of Esculetin

A limited number of studies have been conducted so far for the detection and pharmacokinetic of esculetin. However, researchers around the world have identified several analytical methods that are helpful in detecting esculetin. These methods include the high-performance liquid chromatography (HPLC), Microdialysis with chromatographic analysis, HPLC-diode array UV detection–electrospray ionization tandem mass spectrometry (HPLC-DAD-ESI-MS), gas chromatography and mass spectrometry, and reverse-phase-HPLC (RP-HPLC). With the help of these techniques, the presence of esculetin can be detected, and further studies are conducted to evaluate its in vivo pharmacokinetic profile. The study of absorption, distribution, metabolism, and excretion of a drug over time is known as pharmacokinetics [115].

A study by Li et al. examined the pharmacokinetics profile of esculin and esculetin in rat plasma after receiving 100 mg/kg dose. They used the high-performance liquid chromatography–electrospray tandem mass spectrometry (HPLC-ESI-MS/MS) method to detect esculetin in rat plasma. Pharmacokinetic studies showed that esculin achieved its maximum plasma concentration (C_max_) at 1850.39 ± 129.71 ng/mL, whereas esculetin achieved its C_max_ at 64.62 ± 5.13 ng/mL. Similarly, the time to reach maximum plasma concentration (T_max_) of esculin and esculetin in the plasma of rats was reported to be 10.25 ± 0.03 h and 0.50 ± 0.05 h, respectively [116]. Another group of researchers orally treated the rats with 25 mg/kg dose of esculetin and used the HPLC coupled with ultraviolet detection (XTerra RP 18 column, 85% acetonitrile, 342 nm UV range) and time of flight mass spectrometry (TOF/MS/MS) methods to quantify and identify the esculetin in plasma and tissue of rats. Moreover, the pharmacokinetic studies of esculetin conducted on rats showed the C_max_ and elimination half-lives (T_1/2_) values to be 173.3 ± 25.8 ng/mL and 45 min respectively. The results of their study also highlight the presence of esculetin in liver and kidney tissues up to 180 min after oral treatment [117].

It is well known that increased production of uric acid in the body leads to the development of a condition called hyperuricemia which is the prime cause of gout. The reduced excretion of uric acid causes abnormalities in the renal system [118]. A treatment strategy for gout and hyperuricemia includes the use of *Cortex Fraxini*, which is a well-known traditional Chinese herbal medicine [119]. A study by Wang et al. performed a comparative study on plasma of an orally treated normal and hyperuricemic male Sprague Dawley rats with *Cortex Fraxini* using UPLC-MS/MS method. They showed that the normal rats, after receiving the esculetin therapy at 18 mg/kg, achieved their C_max_ at 369 ± 70 ng/mL, whereas this value was found to be higher in hyperuricemic rats, i.e., 475 ± 82 ng/Ml. Similarly, the T_max_ values were found to be less in normal rats as compared to the hyperuricemic rats, i.e., 0.54 ± 0.25 h and 0.83 ± 0.26 h, respectively. They also reported that there was a gradual decrease in the T_1/2_ of normal rats after receiving the esculetin therapy i.e., 4.01 ± 2.1 h and 4.72 ± 1.4 h, respectively. This leads to a conclusion that the huge difference in the values of studied pharmacokinetic parameters may be the reason for renal dysfunction or impairment in the functioning of the cytochrome P450 metabolic enzyme [120].

A study by Kwak et al. analyzes, for the first time, the bioavailability score of intravenously or orally administrated esculetin (10 mg/kg) in rats. To reveal the pharmacokinetic and bioavailability profile of esculetin, T_1/2_, steady-state distribution volume, clearance time, first-order absorption, and elimination rate constant were identified using WinNonlin^TM^, ADAPT5 software, and the LC-MS/MS method. They showed that values of esculetin in rat plasma were found to be 2.08 ± 0.46 h, 1.81 ±0.52 L/kg, 1.27 ± 0.26 L/h/kg, 0.98 ± 0.18 h^−1^, 2.47 ± 0.28 h^−1^,respectively. Moreover, the bioavailability of esculetin in rat plasma was found to be 19% when orally administrated. This research summarizes that there is a need to ameliorate the bioavailability score of esculetin by increasing its absorption [121]. Likewise, Tsai and colleagues used a microdialysis technique using a microdialysis probe to collect blood from animals receiving esculetin therapy. They placed the blood microdialysis probe into the right jugular vein and adjusted the flow rate of the microinjection pump to 2 pL/min. A 10 mg/kg dose of esculetin was administrated using the intravenous mode of drug delivery, and HPLC combined with UV detection was used to determine the presence of esculetin in blood and bile. A pharmacokinetics study revealed that the distribution half-life (T_1/2α_) of esculetin in blood and bile was 10.3 ± 0.7 min and 35.4 ± 3.8 min, whereas the elimination half-life (T_1/2β_) values of esculetin in blood and bile were54.0 ± 10.9 min and 136.4 ± 24.1 min, respectively. The findings of this study highlighted that the concentration of esculetin in bile was higher than in blood [122].

In another study, Wang et al. attempted to determine the content of aesculetin (also called esculetin) and esculin in orally fed beagle dogs with 0.27 g/kg dose of *L. palustre* extract using the UPLC-ESI-MS/MS method. The pharmacokinetics study on beagle dogs revealed that esculin achieved its C_max_ at 46.75 ± 7.46 ng/mL, and 209.9 ± 57.65 ng/mL for esculetin. Similarly, T_max_ of esculin and esculetin in the plasma of beagle dogs was reported to be 1.32 ± 0.38 h and 1.03 ± 0.27 h, respectively. In addition to these parameters, they also examined the T_1/2_ of esculin and esculetin, which were reported to be 3.43 ± 0.47 h and 4.25 ± 0.18 h, respectively. These results conclude that the absorption speed of esculin is lower than that of esculetin, while the elimination speed of esculetin is lower than that of esculin [123].

Rehman and colleagues administered a 120 mg/kg oral dose of esculetin in rats and used the RP-HPLC method to detect the presence of esculetin in the plasma of orally fed rats. They used an Agilent 1260 infinity HPLC system with 1200 infinity VWD (C18 column: 4.6 mm i.d., 250 mm, and 5 µm). The oven temperature, flow rate, and UV detection wavelength were adjusted to 40 °C, 1.0 mL/min, and 338 nm, respectively. Using this method, the presence of esculetin and esculin was detected to reveal its pharmacokinetics in terms of oral bioavailability in rat plasma. Likewise, the pharmacokinetics of esculin and esculetin in rat plasma were investigated using the RP-HPLC method with UV detection. They showed that C_max_ of esculin and esculetin in the plasma of rats was reported to be 340.3 ± 7.5 ng/mL and 316.5 ± 3.37 ng/mL respectively. On the contrary, the T_max_ of esculin and esculetin were reported to be 0.31 ± 0.04 h and 0.33 ± 0 h. The values of esculin and esculetin for T_1/2_ were found to be 2.23 ± 0.38 h and 3.1 ± 0.5 h, respectively, concluding that esculetin takes more time to eliminate from the body [124].

The metabolism of esculin at the dose of 100 mg/kg in male Sprague Dawley rats was firstly reported by Wang et al. using ultra-high-performance liquid chromatography attached to Fourier-transform ion cyclotron resonance mass spectrometry (UHPLC-FT-ICR-MS). The urine, plasma, bile, and feces samples were collected from rats receiving oral esculetin therapy to know its metabolites. After analysis, a total of 19 metabolites were reported, of which 10 are phase I metabolites and 9 are phase II metabolites. Furthermore, they showed that biotransformation of esculin to esculetin involves deglycosylation. In addition, the metabolic pathway of esculin involves hydrolysis, dehydrogenation, hydroxylation, methylation, dehydrogenation, glucuronidation, sulfation, and glycine conjugation [125].

*Alchemilla speciosa* BUSER is a medicinal herb that belongs to the Rosaceae family. The GC/MS have been extensively used to detect the presence of bioactive compounds in medicinal plant extracts. The presence of esculetin in extract of herb *Alchemilla speciosa* was detected by analytical techniques such as gas chromatography or mass spectrometry [126]. Zhou et al. used the HPLC–DAD–ESI-MS analytical method to detect the presence of coumarins in *Cortex Fraxini*. They used an Agilent 1100 liquid chromatography system (C18 column: 250 mm × 4.6 mm i.d., 5 m) with a diode array detector, autosampler and ESI. The column temperature, detection wavelength, and flow rate were adjusted to 30 °C, 254 nm and 1.0 mL/min, respectively. The results of this study highlighted that there are four types of coumarins present in the *Cortex Fraxini*, and these are esculetin, esculin, fraxin, fraxetin, and escuside [127]; however, further studies focusing on the pharmacokinetic profile of esculetin are still needed.

## 5. Conclusions

A large number of studies have been conducted to reveal the pharmacological and biochemical mechanism of action of coumarin compounds, especially esculetin. As oxidative stress is a known trigger for the onset of many diseases, this coumarin compound scavenges free radicals produced during oxidative stress and thus protects against other diseases such as inflammation, cancer, diabetes, etc. In this review article, we discussed a variety of biochemical modes of action of this molecule, with an emphasis on cancer, oxidative stress, inflammation, arthritis, diabetes, and fatty liver. Free radical scavenging is believed to play a vital role in mediating the antioxidant activity of esculetin and its associated anti-apoptotic effect. Studies have proven that there is a strong connection between oxidative stress and inflammatory mediators. Esculetin is an antioxidant that blocks the redox-dependent NF-κB signaling pathway, which in turn suppresses the synthesis of inflammation-associated mediators, especially cytokines and chemokines. This coumarin compound provides a strong defense against ROS, which helps prevent inflammatory cells from migrating and engaging in other activities. However, there are very few studies on esculetin to prove that it is an anti-inflammatory, so it is concluded that a deeper understanding on the role of esculetin in inflammation and its associated diseases in reducing mortality worldwide is required.

## Figures and Tables

**Table 1:** Mechanism and function of esculetin in treatment of cancer.

Pharmacological Mechanism	Inhibition/Activation/ Downregulation/Upregulation	Model Used	Dosage	Application	Reference
	Cell-cycle arrest at G1-phase Activate ARE pathway and impede binding interactions between Nrf2 and KEAP-1 Attenuate NF-κB pathway	Human PANC-1 cells	100 µM	In vitro	[45]
	Inhibit cell proliferation Induce autophagy by forming autophagic-vesicles Downregulate cyclin D1, D3, DK4 and DK2 Induce cell-cycle arrest at G0/G1-phase Block MEK/ERK phosphorylation by inhibiting Raf/MEK/ERK signaling	Human leukemia cells (HL-60 cells)	20 µM	In vitro	[47]
	Downregulate JNK/ERK signaling	Human leukemia cells (U937 cells)	30 µM	In vitro	[48]
	Downregulate Bcl-2 and NF-κB expressions Induce apoptosis	Benzo[a]pyrene-induced lung carcinogenesis in Swiss-albino mice	50 mg/kg	In vivo	[50]
Anti-cancer	Activate MAPK signaling Activate caspase-3 and 9 and cause apoptosis Release cytochrome c into cytosol Increase mitochondrial membrane depolarization Increase Bax expression	Human colon cancer cells (HT-29 cells)	55 µg/mL	In vitro	[51]
	Suppress SP1, p27, cyclin D1, Mcl-1, survivin expressions Induce apoptosis	Oral squamous cancer (HN22 and HSC4 cells)	20 µg/mL	In vitro	[52]
	Downregulate STAT3 phosphorylation Inhibition of JAK/STAT pathways Induce cell-cycle arrest at G1/S-phase	Laryngeal cancer (Hep2 cells)	2, 10 µM	In vitro, In vivo	[53]
	Cell-cycle arrest at S-phase Elevate caspase-3, 9 expressions Reduce mitochondrial membrane potential Increase Bax expression Downregulate Bcl-2 expression	Hepatocellular carcinoma (C57BL/6 mice were implanted with Hepa1–6 cells and SMMC-7721 cells)	2.24 mM	In vitro, In vivo	[54]
	Suppress IGF-1/PI3K/Akt and IGF-1/MAPK signaling Reduce mitochondrial membrane potential Release cytochrome c from mitochondria Increase Bax, Bcl-2, caspase-3, 9 Expressions	Human gastric cancer (MGC-803 and GES-1 cells)	850 µM	In vitro	[55]
	Inhibit proliferation, migration and invasion of renal cancerous cells Induce cell-cycle arrest at G0/G1 and G2-phase Downregulate cyclin D1, CDK4, CDK6 and c-Myc expressions Increase E-cadherin level by decreasing N-cadherin and vimentin expressions	Renal carcinoma (786-O and SN12-PM6 cells)	200 µg/mL	In vitro	[56]

**Table 2:** Mechanism and function of esculetin in treatment of oxidative stress.

Pharmacological Mechanism	Inhibition/Activation/ Downregulation/Upregulation	Model Used	Dosage	Application	Reference
Antioxidant	Increase phosphorylation of Nrf2 and NQO1 Activate ERK signaling pathways Show protective effect against H_2_O_2_-induced oxidative stress	H_2_O_2_-induced oxidative stress in C2C12 myoblasts cells	5 µM	In vitro	[57]
	Scavenge DPPH, hydroxyl and intracellular ROS Inhibit lipid peroxidation, protein carbonyl and DNA-damage induced by H_2_O_2_	Chinese hamster lung fibroblast cells (V79-4 cells)	10 µg/mL	In vitro	[58]
	Scavenge free radicals Inhibition of lipid peroxidation, AST, ALT and ALP in liver	CCl4-induced acute hepatotoxicity in male Sprague Dawley rats	35 mg/kg	In vivo	[59]
	Activate Nrf2 Increase phosphorylation of ERK signaling and Akt signaling Increase glutathione levels	Amyloid protein-induced oxidative stress and neuronal death in SH-SY5Y cells	20 µM	In vitro	[60]
	Inhibit DPPH, Xanthine oxidase, superoxide radicals Downregulate MMP-1 expression	Oxidative stress in human dermal fibroblasts cells (HDF-cells)	0.6 µg/mL and 2.1 µg/mL	In vitro	[61]
	Inhibit phospho-MEK1, phospho-ERK1/2, phospho-SEK1 and phospho-JNK1/2 along with intracellular Ca^2+^ levels Inhibit MMP-1 expression	H_2_O_2_-induced oxidative stress in Human HaCaT keratinocytes cells	5 µg/mL	In vitro	[62]
	Scavenge hydroxyl radicals and protect DNA from oxidative damage	Lipid-hydroperoxide-induced oxidative damage in human diploid fibroblast cells (TIG-7 cells)	50 µL	In vitro	[63]

**Table 3:** Mechanism and function of esculetin in treatment of inflammation.

Pharmacological Mechanism	Inhibition/Activation/ Downregulation/Upregulation	Model Used	Dosage	Application	Reference
	Downregulate inflammatory cytokines and chemokines (TNF-α, IL-1β, IL-6, CCL2 and iNOS) Inhibit NF-κB, STAT1 and STAT3 expression in macrophage Attenuate IKKα/β, IKBα phosphorylation and p65 levels in LPS-stimulated macrophage Inhibit translocation of p65 from cytoplasm to nucleus in LPS-stimulated macrophage Downregulate phosphorylation of ERK1/2, JNK and p38 levels in macrophage Suppress STAT1 and STAT3 activation in LPS-induced macrophage and sepsis mice	*E. coli*-induced mice sepsis mice and LPS-stimulated macrophage of lung injury (RAW 264.7 cells)	20, 40 and 60 mg/kg	In vitro and in vivo	[67]
	Decrease iNOS and COX-2 level Inhibition of NO and PGE2 production Inhibit TNF-α, IL-1β expression Inhibit LPS-mediated nuclear translocation of NF-κBp65 by suppressing IKβ-α degradation Inhibit ROS generation	LPS-induced inflammation in RAW 264.7 cells	12 µg/mL	In vitro	[68]
	Reverse LTA-induced IkB degradation Reverse NF-κBp65 phosphorylation Increase Nrf2 activity and scavenge DPPH radicals Inhibit NF-κBp65 translocation to nucleus	RAW 264.7 cells	20 µM	In vitro	[69]
	Reduce IL-1β, IL-6, TNF-α in serum and hippocampus Downregulate iNOS and COX-2 in hippocampus Inhibit LPS-induced pIKK-α, pIKK-β, pIKB-α and p-NF-kB65 activation Upregulate p-TrKB protein expression in hippocampus due to activation of BDNF/TrKB signaling pathway, thus exhibit neuroprotective activity	LPS-induced neuro-inflammation in mice and hippocampus protein extract	20, 40 mg/kg	In vivo	[72]
	Increase endocytic activity and augmented NO and iNOS levels in LPS-treated macrophage	LPS-induced inflammation in RAW 264.7 cells and BALB/c mice	80 and 120 µM	In vitro and in vivo	[73]
	Reduce MMP-1 in cartilage Reduce NO and PGE2 in synovial fluid	Knee OA model of rabbit	100 and 200 mg/kg	In vivo	[74]
	Decrease NO, TNF-α and MCP-1 expression Inhibit PPARϒ and CCAAT/enhancer binding protein-α in adipocyte Inhibit iNOS level in macrophage Increase silencing of heme oxygenase	Adipose tissue inflammation model (RAW264.7 cells and 3T3-L1 adipocyte cells)	100 µM	In vitro	[75]
	Inhibit pro-inflammatory cytokines (IL-2, IL-1β, TNF-α, INF-ϒ) in colon Inhibit ROS generation Inhibit MPO and ALP Decrease GSH depletion	TNBS-induced colitis in male Wistar rats and RAW 264.7 cells	5 mg/kg, 100 µM	In vitro and in vivo	[77]
	Increase GSH and serotonin (5-HT) level in brain tissue Decrease TBARS, TNF-α, IL-1β levels in brain tissue	Reserpine-induced fibromyalgia in female Swiss albino mice	100 mg/kg	In vivo	[78]
	Suppress histamine-induced expressions and secretion of IL-6, IL-8, MUC5AC by inhibiting NF-kB signaling pathway Suppress histamine-induced p-p65 expression and p-IKBα degradation	Allergic rhinitis model (Human nasal epithelial cells)	10, 20 and 40 µmol/L	In vivo	[79]
Anti-inflammatory	Reduction in ear swelling Decrease DFE/DNCB-induced scratching Decrease epidermal and dermal thickness Decrease accumulation of mast cells Decrease TNF-α, INF-ϒ, IL-4, IL-13, IL-31, IL-17A-induced phosphorylation of STAT1 and NF-κB (p65) translocation by degrading IKBα	DNCB/DFE—induced atopic skin inflammation model (Female BALB/c mice and Human HaCaT keratinocytes cells)	2, 10, 50 mg/kg and 10 µM	In vitro and in vivo	[80]
	Decrease attenuation of LPS-induced phosphorylation of ERK1/2 and NF-κB expression Protect cells from LPS-induced apoptosis and necrosis Decrease LPS-induced TRAIL, IL-1β, TNFR expression Inhibit LPS-induced MnSOD and GPx Downregulate IL-6, IL-12, VEGF expressions	LPS-induced inflammation in Human retinal pigment epithelial cells (ARPE-19 cells)	5 µM	In vitro	[81]
	Decrease MPO, IL-6, TNF-α, IL-1β expression Inhibit neutrophils infiltration Inhibit LPS-induced RhoA/Rho kinase pathway Block NF-κB activation	LPS-induced acute lung injury (lung epithelial A549 cells and BALB/c mice)	20, 40 mg/kg and 0.1, 1 and 10 µM	In vitro and in vivo	[82]
	Decrease MPO, COX-2, iNOS levels Activate HIF-1in HCT116 cells and increase HIF-1α protein expression Increase secretion of VEGF in HCT116 cells Inhibit HIF-prolyl hydroxylase-2 Enzyme	TNBC-induced colitis (Human colon carcinoma HCT116 cells and Sprague Dawley colitic rats)	100 and 200 µM	In vitro and in vivo	[83]
	Ameliorate skin lesion of psoriatic mice Inhibit CD3+ and CD8+ T-cell infiltration in psoriatic mice skin Decrease Ki67 and K10 mRNA expression Lower effector CD8+ T-cells in lymph nodes and spleen Inhibit NF-κB signaling by suppressing phosphor-IKKα and phosphor-p65 expression Increase CD4+ FOXp3+ Treg frequency in lymph node and spleen Downregulate IL-6, TNF-α, IFN-ϒ, IL-17A, IL-22, IL-23	Imiquimod-induced psoriasis in BALB/c mice	50 and 100 mg/kg	In vivo	[84]

**Table 4:** Mechanism and function of esculetin in treatment of arthritis.

Pharmacological Mechanism	Inhibition/Activation/ Downregulation/Upregulation	Model Used	Dosage	Application	Reference
Anti-arthritic	Inhibit IL-1α-induced release of proteoglycan in cartilage Downregulate proMMP-1 and MMP-3 expression in cartilage Inhibit matrix degradation in rabbit joints	OA rabbit model	10–100 µM	In vivo	[95]
	Suppress proteoglycan depletion in chondrocyte Inhibit MMP production Downregulate pro-MMP 1 and pro-MMP3 in rabbit chondrocyte	Rabbit articular chondrocyte	100 µM	In vivo	[96]
	Decrease leukotriene B4 level in plasma	AIA in male Lewis rats	10 mg/kg	In vivo	[97]
	Decline paw volume Prevent swelling, bone and cartilage destruction Downregulate Cat-D, ACP, ALP and TRAP bone degrading enzymes Inhibit endogenous generation of ROS and TNF-α, IL-1β, IL-6, COX-2 and PGE2 level Increase ALT and AST level Inhibit NF-κB and Akt signaling pathway Restore SOD, CAT and GST enzyme	AIA in adult Wistar rats	50 mg/kg	In vivo	[98]
	Inhibition of proteoglycan and collagen resorption Inhibit IL-1α + oncostatin M stimulated resorption and decreased the MMP-1 level Reduce IL-1α + oncostatin M induced expressions of MMP-1, MMP-3 and MMP-13	Transformed human chondrocyte cells (T/C28a4 cells)	66 µM, 100 µM and 50 µmol/L	In vitro	[99]

**Table 5:** Mechanism and function of esculetin in treatment of diabetes and complications.

Pharmacological Mechanism	Inhibition/Activation/ Downregulation/Upregulation	Model Used	Dosage	Application	Reference
Anti-diabetic and its complications	Restore level of antioxidant enzymes (GST, COD, CAT, GPx) Increase plasma insulin in diabetic rats Decrease blood glucose in diabetic rats Decrease TBARS, lipid hydroperoxides and conjugated dienes in liver and kidney Increase vitamin C, tocopherol and reduce glutathione in kidney tissues of diabetic rats	STZ-induced diabetes in male albino rats	40 mg/kg	In vivo	[103]
	Prevent increase in angiotensin II type I receptor and angiotensin II type 2 receptor expression Improve insulin sensitivity and reduce systolic blood pressure Attenuate vascular hyper-responsiveness to Angiotensin II and impair acetylcholine-mediated relaxation Decrease TGF-β and KEAP-1 expression	HFD + STZ-induced hyperinsulinemia and hyperglycemia in male Wistar rats	50 and 100 mg/kg	In vivo	[104]
	Decrease blood glucose, urea nitrogen, plasma creatinine Increase plasma albumin level Attenuate downregulation of PPARϒ in diabetic kidney and blocks TGF-β1-mediated fibronectin expression Attenuate decrease in mono-methylation (k4) and acetylation of histone H3 in diabetic kidney Decrease Bmp6 expression and increase Mmp13 expression in diabetic kidney	STZ-induced diabetic nephropathy in Sprague Dawley rats	50 and 100 mg/kg	In vivo	[105]
	Attenuate alteration in RAS, KEAP-1 and cell proliferation (Ki67) Decrease systolic blood pressure, plasma glucose, triacylglycerol and total cholesterol in insulin resistance and type 2 diabetic rats Prevent cardiac hypertrophy and cardiac fibrosis in diabetic rats Reduce AT1R, A2TR, KEAP, Ki67 and increase ACE2 expression in insulin resistance and type 2 diabetic rats Attenuate H2AK119Ub and H2BK12OUb level in heart tissue of insulin resistance and type 2 diabetic rats	STZ-induced type diabetes and diabetic cardiomyopathy	50 and 100 mg/kg	In vivo	[106]
	Improve insulin sensitivity, hyperglycemia, and renal dysfunction Increase SOD1, GSH level and decrease TBARS levels in diabetic rats Increase angiotensin I converting enzyme 2 (ACE2) Decrease angiotensin II receptor type I and angiotensin II converting enzyme Decrease MCP-1 and TGF-β expressions in diabetic kidney Decrease H2AK119Ub expression in diabetic kidney	HFD + STZ-induced diabetic nephropathy	50 and 100 mg/kg	In vivo	[107]

**Table 6:** Mechanism and function of esculetin in treatment of hepatic disease.

Pharmacological Mechanism	Inhibition/Activation/ Downregulation/Upregulation	Model Used	Dosage	Application	Reference
Anti-hepatic	Decrease plasma triglyceride, cholesterol, and insulin levels Increase AST, and ALT and prevent hepatic fibrosis Inhibit lipid peroxidation and increase GSH level in HFD-fed rats Increase FOXO1 phosphorylation in liver tissue of HFD-fed rats Prevent accumulation of extracellular matrix protein in the liver by reducing TGF-β expression	HFD-induced fatty liver in male Wistar rats	50 and 100 mg/kg	In vivo	[108]
	Downregulate lipid synthesis genes (Fasn, Dgat2, pap) and inflammatory genes (TLR4, Myd88, NF-kB, TNF-α, IL-6, and MCP-1) Increase SOD level and inhibit lipid peroxidation	HFD-induced non-alcoholic fatty liver in diabetes in C57BL/6N mice	0.01% *w*/*w*	In vivo	[109]
	Increase phosphorylation of AMPK-α (Thr172) and ACC (Ser79) Decrease SREBP1c and FAS Activate AMPK signaling pathway	Free fatty acid-induced lipid accumulation in Human HepG2 cells	25, 50 and 100 µM	In vitro	[110]

## Data Availability

Not applicable.

## Abbreviations

DNA: Deoxyribonucleic acid; JNK: c-Jun-N-terminal kinase; H_2_O_2_: Hydrogen peroxide; ROS: Reactive oxygen species; RA: Rheumatoid arthritis; Nrf2: Nuclear factor erythroid-2-related factor; NF-κB: Nuclear factor-kappa B; MAPK: Mitogen-activated protein kinase; ERK: Extracellular signal-regulated kinase; Bcl-2: B-cell lymphoma-2; JAK/STAT: Janus kinase-signal transducer and activator of transcription; Bax: B-cell lymphoma-2-associated x protein; IGF1: Insulin-like growth factor 1; PI3K: Phosphoinositide-3-kinase; Akt: Protein kinase B; c-Myc: Cellular myelocytomatosis; SP1: Specificity protein 1; ALP: Alkaline phosphatase; AST: Aspartate transaminase; ALT: Alanine transaminase; MMP: Matrix metalloproteinase; NO: Nitric oxide; TNF-α: Tumor necrosis factor-α; IL-1β: Interleukin-1β; iNOS: Inducible nitric oxide synthetase; LPS: Lipopolysaccharide; NOS: Nitric oxide synthase; COX: Cyclooxygenase; PGE: Prostaglandins; PPAR: Peroxisome proliferator-activated receptor; IFN: Interferon; GSH: Reduced glutathione; MPO: Myeloperoxidase; VEGF: Vascular epidermal growth factor; MnSOD: Manganese superoxide dismutase; HIF: Hypoxia-inducible factor: SOD: Superoxide dismutase; CAT: Catalase; GST: Glutathione-s-transferase; STZ: Streptozotocin; GPx: Glutathione peroxidase; TBARS: Thiobarbituric acid reactive substances; TGF-β: Transforming growth factor-β; HFD: High fat diet; ACE2: Angiotensin converting enzyme 2; FOXO1: Fork head box protein O1; SREBP1c: Sterol regulatory element-binding protein-1c; AMPK: Adenosine monophosphate-activated protein kinase; PPSA: Phospoenolpyruvate synthetase; aroG: Deoxyphosphoheptonate dehydrogenase; pheA: Prehenate dehydratase; tyrB: Phenylalanine aminotransferase; TAL: Tyrosine amino lyase; 4CL: 4-Coumaroyl-CoA ligase; C3H: Coumarate-3-hydroxylase; F6′H: Feruloyl CoA6′-hydroxylase; HPLC: High performance liquid chromatography; HPLC-DAD-ESI-MS: HPLC-diode array UV-detection-electrospray ionization tandem mass spectrometry; RP-HPLC: Reverse phase-HPLC; C_max_: Maximum plasma concentration; T_max_: Time to reach maximum plasma concentration; T_1/2_: elimination half-lives.
